# Postvoid Residual Urine as a Clue to the Diagnosis of Acute Bacterial Prostatitis: A Case Report

**DOI:** 10.7759/cureus.116448

**Published:** 2026-09-17

**Authors:** Yoshihiro Kawaguchi

**Affiliations:** 1 Department of Urology, Morodomi Central Hospital, Saga-shi, JPN

**Keywords:** acute bacterial prostatitis, computed tomography, escherichia coli, postvoid residual urine, voiding dysfunction

## Abstract

Acute bacterial prostatitis (ABP) typically presents with fever and urinary symptoms; however, its diagnosis can be challenging in patients who lack characteristic findings such as perineal pain or prostatic tenderness. Although postvoid residual (PVR) urine may be associated with voiding dysfunction in ABP, its potential role as a diagnostic clue has received little attention. A 44-year-old man presented with a 2-day history of fever ranging from 38 to 39°C, irritative urinary symptoms, back pain, and difficulty initiating urination. At presentation, the patient was afebrile and had no perineal tenderness. Noncontrast computed tomography (CT) performed approximately 1 min after urination revealed an estimated PVR volume of 170 mL. The CT also showed an enlarged prostate with irregular margins and a low-density area. Laboratory tests showed a white blood cell count of 17,700/μL, a C-reactive protein level of 7.01 mg/dL, and 3+ leukocytes on urinalysis. Urine culture subsequently yielded *Escherichia coli*. Based on the clinical, imaging, and laboratory findings, ABP was diagnosed, and antibiotic therapy was administered for 28 days. Following treatment, inflammatory markers and urinary findings improved, and the follow-up PVR volume was 36 mL when measured 10 min after urination. In this case, PVR volume measurement immediately after urination provided a clue to the presence of voiding dysfunction associated with ABP, despite the absence of typical clinical findings. Furthermore, the reduction in PVR volume following treatment was temporally associated with improvement in inflammatory findings and urinary symptoms, although a causal relationship cannot be established. In patients with urinary symptoms and suspected ABP despite the absence of characteristic clinical findings, assessment of PVR volume may provide additional information regarding associated voiding dysfunction and support the overall clinical assessment.

## Introduction

Acute bacterial prostatitis (ABP) is a bacterial infection of the prostate that commonly presents with fever, urinary symptoms, and perineal pain. Urinary tract obstruction and urinary retention may complicate ABP, and the European Association of Urology (EAU) guidelines recognize urinary retention as a potential complication of ABP that may require temporary urinary drainage [1]. The diagnosis and management of ABP can be challenging and require a comprehensive assessment of clinical symptoms, physical findings, urinalysis, and microbiological findings [2,3].

Furthermore, a multicenter retrospective study of 371 patients with acute prostatitis highlighted that patients frequently present with bladder outlet obstruction and require evaluation of voiding function, suggesting that voiding dysfunction associated with ABP extends beyond complete urinary retention [4].

However, the clinical significance of postvoid residual (PVR) in patients with ABP and its potential role in the diagnostic assessment of ABP and posttreatment evaluation of voiding function remain unclear.

We report a case of ABP in a patient who lacked characteristic perineal pain or prostatic tenderness. A PVR volume of approximately 170 mL was detected on non-contrast computed tomography (CT) performed immediately after urination, indicating incomplete bladder emptying and contributing to the overall clinical assessment of suspected ABP. In this case, the urinary symptoms improved, and the PVR volume was 36 mL at follow-up after antimicrobial therapy. This case highlights the importance of paying attention not only to urinary retention but also to incomplete bladder emptying in patients with ABP, and suggests that PVR measurement may be useful for assessing associated voiding dysfunction and changes in bladder emptying following treatment.

## Case presentation

A 44-year-old man presented with a 2-day history of fever ranging from 38°C to 39°C, irritative urinary symptoms, back pain, and difficulty initiating urination. He was afebrile at presentation. Acute pyelonephritis, urinary tract stones, and acute bacterial prostatitis (ABP) were considered in the differential diagnosis, and non-contrast CT of the abdomen and pelvis was performed to identify the underlying cause (Figure 1).

**Figure 1:**
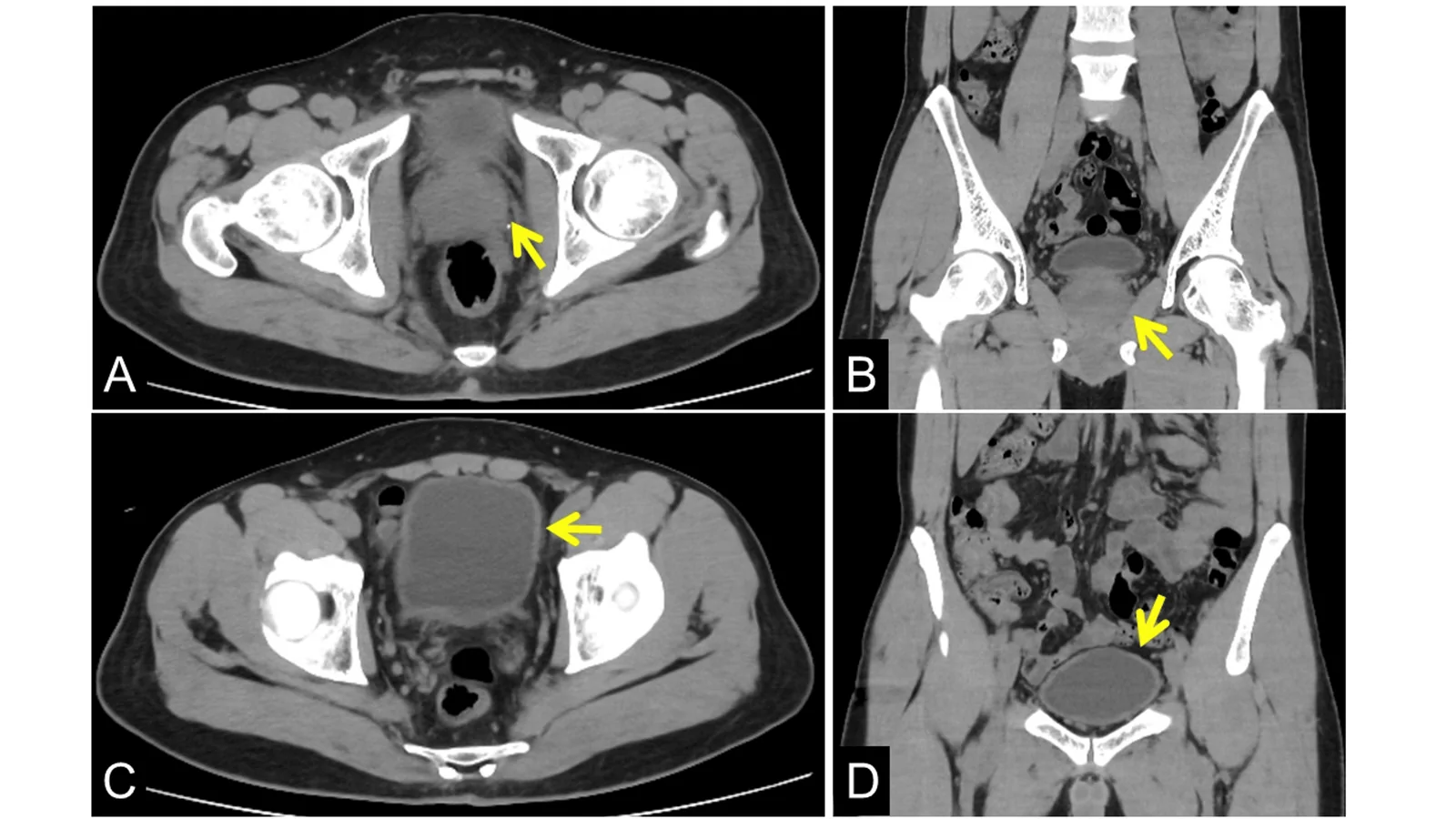
Non-contrast computed tomography findings at the initial visit. A, B: Prostate findings. (A) Axial image showing the enlarged prostate with irregular margins. (B) Coronal image showing an irregular prostate margin and a low-density area within the prostate. The prostate is indicated by yellow arrows in A and B. C, D: Bladder findings. (C) Axial image showing approximately 170 mL of residual urine despite voiding immediately before CT. (D) Coronal image showing residual urine within the bladder. The bladder is indicated by yellow arrows in C and D.

At presentation, the patient was afebrile. Digital rectal examination (DRE) was omitted to avoid the risk of inducing bacteremia and severe pain associated with acute prostatic inflammation. Instead, gentle perineal palpation over clothing was performed, which revealed no tenderness. Approximately 1 min after urination, non-contrast CT revealed a substantial volume of residual urine in the bladder. The PVR volume was estimated using the INFINITT PACS. On the axial CT image, the transverse bladder diameter was 7.61 cm, and on the sagittal CT image, the bladder dimensions were 7.35 × 5.80 cm. The bladder volume was calculated using the ellipsoid formula (length × width × height × 0.52), yielding 169.5 mL, which was rounded to approximately 170 mL. The same CT scan showed an enlarged prostate with irregular margins and a low-density area. No urinary tract stones or other clear upper urinary tract abnormalities were identified.

Laboratory tests showed an elevated white blood cell (WBC) count of 17,700/μL and a C-reactive protein (CRP) level of 7.01 mg/dL. Urinalysis revealed 3+ leukocytes and positive occult blood, whereas urine sediment examination showed >100 WBCs/high-power field (HPF) and 10-19 red blood cells (RBCs)/HPF (Table 1).

**Table 1:** Clinical course and changes in laboratory findings, urinary findings, and antimicrobial therapy. WBC: white blood cell; CRP: C-reactive protein; RBC: red blood cell; PVR: post-void residual; CTRX: ceftriaxone; LVFX: levofloxacin. *PVR was estimated using non-contrast CT performed approximately 1 min after urinalysis. †PVR was measured using ultrasonography 10 min after voiding.

Parameter	Initial visit	Day 6	Final follow-up	Reference range
WBC (/μL)	17,700	6,300	5,500	3500-9800
CRP (mg/dL)	7.01	0.78	0.02	0–0.30
Urinary WBC (dipstick)	3+	−	−	Negative
Urinary sediment WBC (/HPF)	>100	1–4	1–4	0–4
Urinary sediment RBC (/HPF)	10–19	1–4	10–19	0–4
PVR (mL)	~170*	—	36†	
Antimicrobial therapy	CTRX	CTRX → LVFX	—	

Serum PSA was not measured at the initial visit because it was not considered necessary for the immediate diagnostic assessment. Blood cultures were not obtained because the patient was afebrile and clinically stable at presentation. Based on the history of high-grade fever and urinary symptoms, marked inflammatory response, pyuria, substantial PVR, and characteristic prostate findings on CT, ABP was suspected. Acute pyelonephritis and urinary tract stones were considered less likely because no urinary tract stones or other clear upper urinary tract abnormalities were identified on CT. Urine culture subsequently yielded Escherichia coli (1+), further supporting the diagnosis of ABP.

Treatment with ceftriaxone (CTRX) was initiated. Six days after treatment initiation, the WBC count decreased to 6,300/μL and the CRP level decreased to 0.78 mg/dL, while urinary leukocytes became negative. Urine sediment also decreased to 1-4 WBCs/HPF and 1-4 RBCs/HPF, and the patient’s urinary symptoms improved. Antimicrobial susceptibility testing revealed intermediate susceptibility to piperacillin (PIPC) and susceptibility to CTRX and levofloxacin (LVFX). Based on the urine culture and antimicrobial susceptibility results, treatment was switched to oral LVFX and continued. Antimicrobial therapy was administered for 28 days.

Urinary symptoms, inflammatory markers, urinary findings, and PVR were reassessed at the final follow-up. The WBC count had decreased to 5,500/μL and the CRP level to 0.02 mg/dL. Urinary leukocytes remained negative, while urinary sediment examination showed 1-4 WBCs/HPF. The PVR measured by ultrasonography 10 min after urination was 36 mL, and his urinary symptoms had improved (Figure 2). Therefore, antimicrobial treatment was discontinued.

**Figure 2:**
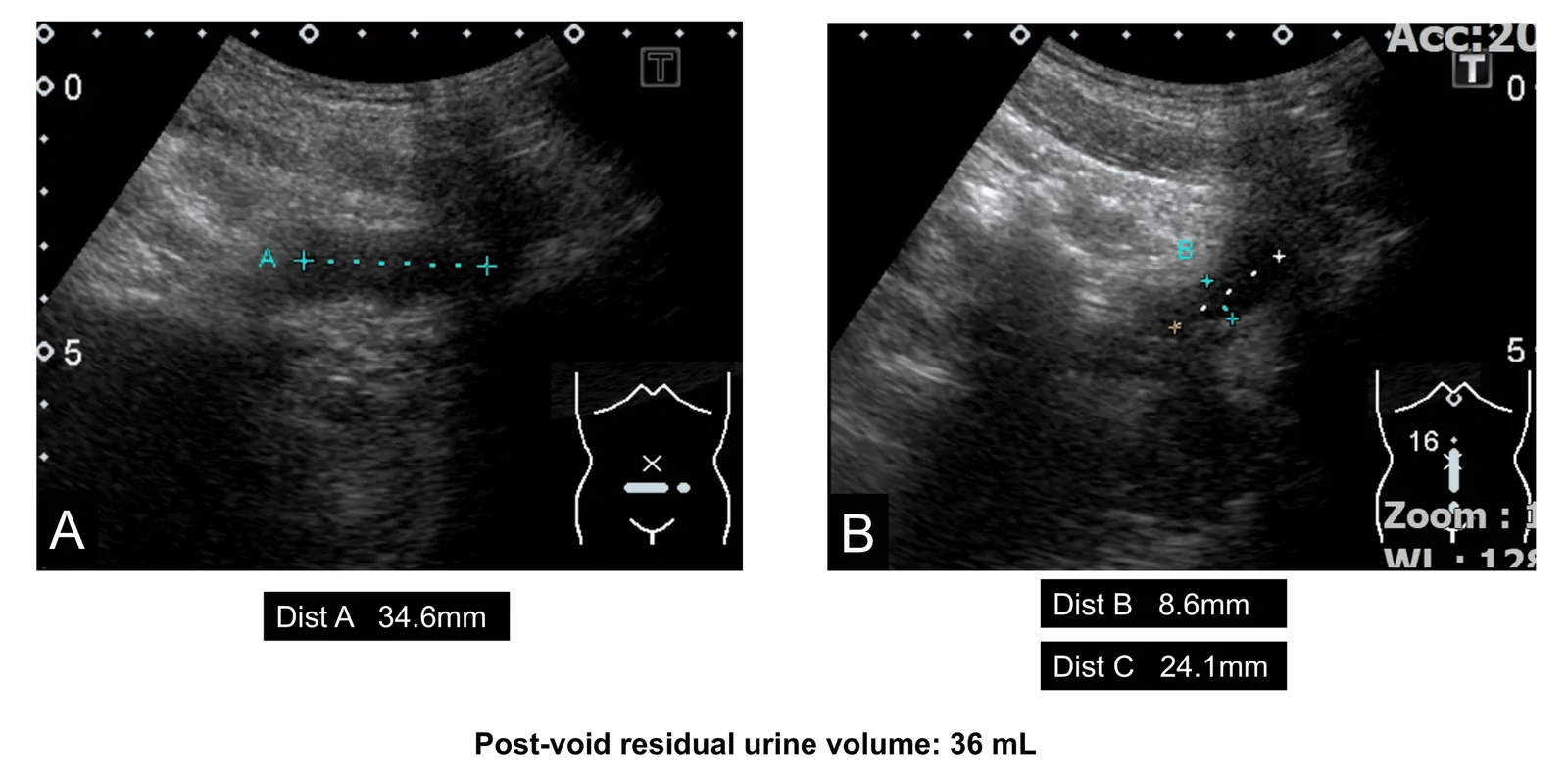
Ultrasonographic measurement of postvoid residual urine volume after antimicrobial therapy. (A) Transverse view. (B) Sagittal view. The postvoid residual urine volume was 36 mL, measured 10 min after voiding.

## Discussion

This case highlights two important aspects of voiding dysfunction associated with ABP. First, the detection of an elevated PVR volume immediately after urination provided additional information regarding voiding dysfunction and contributed to the overall clinical assessment of suspected ABP in a patient without typical clinical findings. Second, the temporal association between improvement in urinary symptoms and inflammatory markers and the lower PVR volume observed at follow-up raises the possibility that inflammatory changes associated with ABP may have contributed to the initial voiding dysfunction.

ABP is characterized by fever, urinary symptoms, and perineal pain, and may be complicated by bladder outlet obstruction or urinary retention. The EAU guidelines note that acute urinary retention can complicate ABP, requiring temporary catheterization in severe cases [1]. Additionally, multicenter observational data of 371 patients with acute prostatitis demonstrated a high frequency of voiding difficulty and bladder outlet symptoms requiring clinical evaluation, supporting the concept that non-retentive voiding dysfunction and incomplete bladder emptying are relevant clinical features of ABP [4].

The diagnosis of ABP is challenging. Lam et al. noted that high-quality evidence regarding the interpretation of clinical and laboratory findings in bacterial prostatitis is limited and emphasized the importance of integrating clinical findings with microbiological testing [2]. In this case, the patient was afebrile at presentation and had no perineal pain or prostatic tenderness. However, a PVR volume of approximately 170 mL was detected on CT approximately 1 min after urination, indicating voiding dysfunction. In addition, prostate enlargement with irregular margins and a low-density area, elevated inflammatory markers, increased urinary leukocytes, and a positive urine culture for Escherichia coli supported the diagnosis of ABP. Thus, PVR volume assessment may provide additional information regarding associated voiding dysfunction and contribute to the overall clinical assessment when ABP is suspected.

Voiding dysfunction associated with ABP may result from prostatic inflammation and edema, which can increase urethral resistance and potentially lead to difficulty voiding or urinary retention [1,4]. In this case, the PVR volume was approximately 170 mL when measured 1 min after urination at the initial visit and 36 mL when measured 10 min after urination at follow-up after antimicrobial therapy. The lower PVR volume observed at follow-up was temporally associated with improvement in inflammatory markers and urinary symptoms; however, the difference in measurement method and timing after urination precludes direct comparison of the two PVR values. The initial PVR was estimated using non-contrast CT approximately 1 min after urination, whereas the follow-up PVR was measured by ultrasonography 10 min after urination. Therefore, the observed difference should be interpreted cautiously and cannot be considered definitive evidence of improvement in voiding function. Nevertheless, the temporal association between clinical improvement and the lower PVR value raises the possibility that the initial voiding dysfunction may have been associated with the acute inflammatory process.

The clinical presentation of ABP is heterogeneous, and its diagnosis may require the integration of multiple clinical and laboratory findings. Similarly, Kinoshita et al. reported a case of ABP in which the diagnosis was established by integrating clinical findings with urinalysis and urine culture results [5]. Although the patient's age and clinical background differed from those of the present case, the case supports the importance of integrating multiple findings rather than relying on a single symptom or finding.

This study has several limitations. First, we could not establish whether the increased PVR volume was directly caused by inflammatory changes associated with prostatitis. Second, uroflowmetry and comprehensive lower urinary tract function testing were not performed before treatment; therefore, underlying bladder outlet obstruction or pre-existing voiding dysfunction cannot be completely excluded. In addition, the initial PVR volume was estimated using CT, whereas the posttreatment PVR was measured using ultrasonography. Differences in measurement methods and timing after urination should be considered. Nevertheless, the temporal relationship between the initially elevated PVR volume, the subsequent diagnosis and treatment of ABP, the improvement in urinary symptoms, and the lower PVR volume observed at follow-up raises the possibility of an association between ABP and the initial voiding dysfunction.

Taken together, this case suggests that attention should be paid not only to urinary retention, but also to incomplete bladder emptying, as reflected by an elevated PVR volume, in patients with ABP. In patients with urinary symptoms and suspected ABP despite the absence of characteristic findings such as perineal pain or prostatic tenderness, PVR assessment may provide additional information regarding associated voiding dysfunction and support the overall clinical assessment. Serial PVR assessments may also be useful for monitoring changes in ABP-associated voiding dysfunction. Further studies are required to determine the prevalence, clinical significance, and temporal relationship between elevated PVR and ABP.

## Conclusions

This case suggests that PVR volume assessment may provide additional information regarding associated voiding dysfunction and support the overall clinical assessment of suspected ABP in patients with urinary symptoms but without typical perineal pain or prostatic tenderness. In this patient, a PVR volume of approximately 170 mL was detected immediately after urination, whereas the follow-up PVR volume was 36 mL after antimicrobial therapy. Although a causal relationship between ABP and voiding dysfunction cannot be established from a single case, serial PVR assessments may provide additional information regarding voiding dysfunction in patients with suspected ABP. Further studies are required to determine the prevalence, clinical significance, and temporal relationship between elevated PVR and ABP.

## References

[1] Kranz J, Cai T, Geerlings S (2026). European Association of Urology guidelines on urological infections: summary of the 2026 guidelines [Epub ahead of print]. Eur Urol.

[2] Lam JC, Lang R, Stokes W (2023). How I manage bacterial prostatitis. Clin Microbiol Infect.

[3] Borgert BJ, Wallen EM, Pham MN (2025). Prostatitis: a review. JAMA.

[4] Etienne M, Chavanet P, Sibert L (2008). Acute bacterial prostatitis: heterogeneity in diagnostic criteria and management. Retrospective multicentric analysis of 371 patients diagnosed with acute prostatitis. BMC Infect Dis.

[5] Kinoshita K, Suzuki M, Koketsu M, Fukasawa T, Kubota T (2024). Acute bacterial prostatitis in a 12-year-old boy without any underlying disease. Cureus.

